# Improving Homology-Directed Repair in Genome Editing Experiments by Influencing the Cell Cycle

**DOI:** 10.3390/ijms23115992

**Published:** 2022-05-26

**Authors:** Svetlana A. Smirnikhina, Milyausha I. Zaynitdinova, Vasilina A. Sergeeva, Alexander V. Lavrov

**Affiliations:** Research Centre for Medical Genetics, Laboratory of Genome Editing, 115478 Moscow, Russia; milyazayn@gmail.com (M.I.Z.); tracytheplane@gmail.com (V.A.S.); alexandervlavrov@gmail.com (A.V.L.)

**Keywords:** cell cycle, CRISPR-Cas9, mitogens, HDR, p53

## Abstract

Genome editing is currently widely used in biomedical research; however, the use of this method in the clinic is still limited because of its low efficiency and possible side effects. Moreover, the correction of mutations that cause diseases in humans seems to be extremely important and promising. Numerous attempts to improve the efficiency of homology-directed repair-mediated correction of mutations in mammalian cells have focused on influencing the cell cycle. Homology-directed repair is known to occur only in the late S and G2 phases of the cell cycle, so researchers are looking for safe ways to enrich the cell culture with cells in these phases of the cell cycle. This review surveys the main approaches to influencing the cell cycle in genome editing experiments (predominantly using Cas9), for example, the use of cell cycle synchronizers, mitogens, substances that affect cyclin-dependent kinases, hypothermia, inhibition of p53, etc. Despite the fact that all these approaches have a reversible effect on the cell cycle, it is necessary to use them with caution, since cells during the arrest of the cell cycle can accumulate mutations, which can potentially lead to their malignant transformation.

## 1. Introduction

Genetic engineering allows precise manipulation of the genome. The available systems make it possible to perform site-specific modification of the genome, which can be used to analyze the functions of genes, create cellular and animal models of diseases, and develop new methods of treatment. Zinc finger nuclease (ZFN) [1,2] and transcriptional activator-like effector nuclease (TALEN) [3] technologies employ sequence-specific DNA-binding modules to induce DNA damage and increase gene targeting efficiency, whereas a method based on clustered regularly interspaced palindromic repeats (CRISPR) with CRISPR-associated protein 9 (Cas9) [4] utilizes RNA guides.

As a result of the action of ZFNs, TALENs, or Cas9, a DNA double-stranded break (DSB) occurs, which can be repaired by one of three major mechanisms: microhomology-mediated end joining (MMEJ), non-homologous end joining (NHEJ) or homology-directed repair (HDR) [5]. The NHEJ pathway has a significant drawback: in the process of ligation of DNA ends, insertions or deletions (indels) can be introduced into the DNA sequence in the DSB region [6]. The HDR pathway requires a donor molecule (usually from a sister chromatid) to recombine to restore the correct DNA sequence [7]. Such precise modifications are desirable for targeted genome engineering. It should be noted that the term ‘HDR’ is often used to refer to both homology-directed repair (HDR), which is activated when a single-stranded oligodeoxyribonucleotide (ssODN) is used as a template and is regulated by the BRCA1–RAD52 axis, and homologous recombination (HR), which is activated when a double-stranded donor is used as a template and is regulated by the BRCA2–RAD51 axis. In this article, we use the term ‘HDR’ for both cases. MMEJ is a variant of the alternative NHEJ and is based on the occurrence of microhomology of sequences ranging in length from 5 to 25 base pairs. This DSB repair pathway is classified as highly error-prone [5].

The cell cycle consists of several phases: the synthetic (S-phase), mitotic (M-phase), and growth (G1 and G2) phases. The transition between these phases is regulated by specific factors, the main factors of which are cyclin-dependent kinases (CDKs). Their activity is influenced by various external (inhibitors) and internal (for example, cell size or DNA damage) factors [8]. The key points of cell cycle advancement and arrest and the possibility of influencing these processes are discussed below.

The choice of the DNA repair pathway in mammalian cells is often determined by the phase of the cell cycle. NHEJ acts during all phases of the cell cycle and generally joins two DSB ends by direct ligation [9], whereas HDR is restricted to the late S and G2 phases, when DNA replication is completed and sister chromatids are available to serve as repair templates [10]. There is evidence that NHEJ inhibition (by chemical substances or siRNA/shRNA) could improve the rate of HDR [11,12]. Another approach is to increase the HDR rate by stimulation of key HDR factors, such as RAD51, CtIP, or CDK1, by small molecules or CRISPRi (e.g., by suppression of KU80 expression) [12]. However, the mechanisms of action of many small molecules remain unknown.

On the other hand, enrichment for cells in the late S and G2 phases of the cell cycle may be beneficial. Cell cycle synchronization, the use of mitogens, or the selection of cells in a particular phase by flow cytometry may increase the proportion of cells with DSBs repaired by HDR. In this review, we surveyed the main approaches for influencing the cell cycle that can be used to increase the number of knock-in events in genome editing experiments.

## 2. Antimicrotubular Agents (Antimitotic Agents)

Microtubules (MTs) are cytoskeletal structures composed of α- and β-tubulin dimerized subunits. MTs form hollow, cylindrical, filamentous structures characterized by a highly dynamic behavior [13]. MTs play a key role in cell growth and division, cell motility, and cell shape; in addition, they regulate intracellular trafficking [14]. MTs exist in a dynamic equilibrium that is characterized by the non-covalent binding of tubulin dimers to a growing MT and subsequent depolymerization with the formation of dimers again [15]. MTs are crucial for the mitotic spindle, the disruption of which leads to the inability to correctly segregate their chromosomes during cell division [16]. Both inhibition of tubulin polymerization/stabilization and MT disassembly interfere with the dynamic equilibrium of cells. Cells treated with antimitotic agents have condensed chromosomes, no nuclear envelope, and a deformed or absent mitotic spindle [17].

Antimicrotubular agents are substances that interfere with the functioning of microtubules and thus inhibit cell division. This group includes a wide variety of natural, synthetic, and semisynthetic substances with different chemical structures. They can be divided into two subgroups on the basis of their mode of action. The first group comprises inhibitors of microtubule assembly; these substances bind to the CLC (colchicine) site or the vinca domain (vinca alkaloids) or alkylate tubulin sulfhydryl groups. The second group comprises stabilizers of microtubules; they bind to polymerized tubulin with a high affinity [18].

Examples of the use of these substances in genome editing experiments are summarized in Table 1 and are discussed in detail below.

### 2.1. Nocodazole

Nocodazole is a CLC site tubulin polymerization inhibitor with a low affinity for βIII-tubulin [25]. It is widely used to arrest cells in the G2/M phases [26] and can be used in genome editing technologies for HDR improvement. Treatment with nocodazole leads to a reversible cell cycle synchronization in various cell cultures [19,22] but not in primary neonatal fibroblasts or embryonic stem cells [19]. In porcine fetal fibroblasts, nocodazole (1 µg/mL) resulted in a 2.8-fold increase in HDR (up to 29.6%) in GFP knock-in experiments using CRISPR-Cas9 [24]. In HEK293T, nocodazole increased the HDR rate up to 6-fold with CRISPR-Cas9 and ssODN as a template, depending on the target locus. In this study, cells (<70% confluency) were treated with nocodazole (200 ng/mL) for 17 h [19]. In induced pluripotent stem cells (iPSCs), the HDR rate increased by only 1.7 times (from 13 to 22%) [22]. Yang et al. [23] demonstrated that up to 80% of human pluripotent stem cells (hPSCs) were in the G2/M phase after treatment with nocodazole (16 h incubation with nocodazole at 1 μg/mL followed by 1 h release) compared with the majority (58.1%) of untreated cells in the G1 phase. The HDR rate increased by 3.5 times (up to 1.5%) without cell selection and up to 78% using Cas9 nickase and antibiotic selection. Knock-in was also 3–6 times higher for ZFNs and TALENs [23]. The advantages of nocodazole are the reversibility of cell cycle synchronization and the absence of effect on pluripotency of stem cells. However, there are no data regarding the apoptotic effect of nocodazole in mammalian cells.

Zhang et al. (2017) used a combination of nocodazole with cyclin D1 (CCND1), which induces the cell cycle transition from the G0/G1 to the S phase [27]. This approach allowed the researchers to double the HDR efficiency to reach 30% in iPSCs. However, Yan et al. [20] found no additional increase in HDR in cells treated with nocodazole and selected on puromycin compared with cells selected on puromycin alone. Thus, in some cases, nocodazole does not have a positive effect on HDR.

### 2.2. ABT-751

ABT-751 is a sulfonamide that interacts with the CLC binding site of β-tubulin, inhibiting microtubule polymerization and causing cell cycle arrest in the G2/M phase as well as apoptosis [28,29]. In one study, Yang et al. demonstrated that up to 80% of hPSCs were in the G2/M phase after treatment with ABT-751 (0.37 μg/mL, 16 h, no release) and observed a 3.1-fold increase in HDR rate in various hPSC lines (up to 1.35%) in experiments with CRISPR-Cas9 without selection. Cell cycle synchronization was reversible and did not affect pluripotency [23].

### 2.3. Vinca Alkaloids

Vinca alkaloids are natural products derived from Vinca plants [30]. Vincristine and vinblastine [26] were among the earliest anti-tumor agents recognized as tubulin polymerization inhibitors. The mechanism of tubulin assembly inhibition was discovered in 1965. Vinca alkaloids arrest the cell cycle in the G2/M phase by quickly and reversibly binding to the β-tubulin subunit in the region called the vinca domain [30]. Rahman and colleagues [21] demonstrated that vinblastine induced G2 cell arrest in immortalized cell cultures HeLa, HT-1080, and U-2 OS. The HDR rate increased up to 7-fold in HeLa cells after treatment with vinblastine (4 h, 40 nM) in experiments with meganuclease I-SceI and ZFNs; however, vinblastine is highly cytotoxic. In experiments with meganuclease I-SceI and ZFNs, there was no increase in HDR in umbilical cord-derived mesenchymal stem cells.

## 3. Cell Cycle-Dependent Expression of Cas9

**Figure 1:**
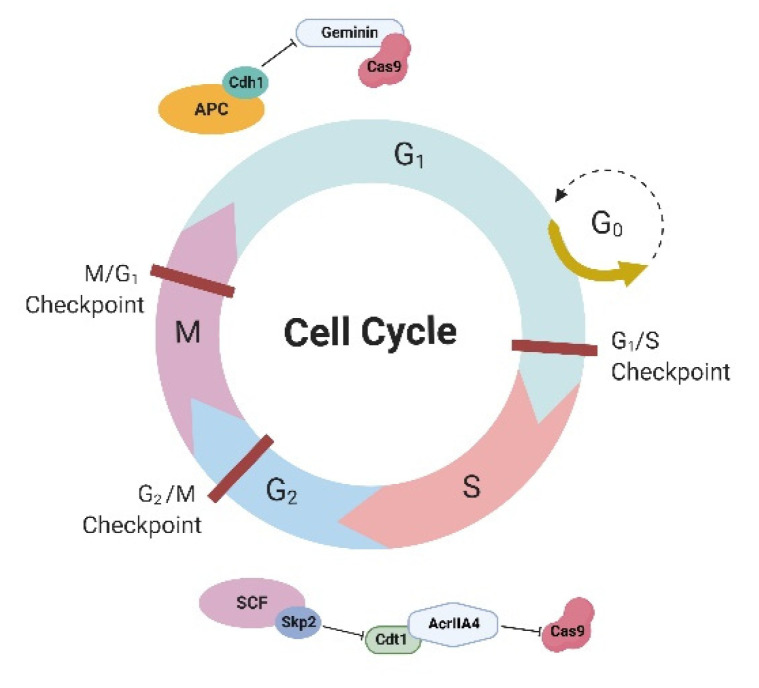
Factors providing Cas9 expression in a cell cycle-dependent manner. Fusion of Cas9 with geminin resulted in lower expression of Cas9 in G1 and higher expression in S/G2/M phases because of degradation by APC-Cdh1. Since Cdt1 is active in the G1 phase, the AcrIIA4-hCdt1 complex inhibits Cas9 in the G1 phase; in the S/G2 phases, the complex undergoes proteolysis by the SCF-Skp2 complex and releases Cas9 activity. APC—anaphase-promoting complex, Cdh1—Cadherin-1, hCdt1—human chromatin licensing and DNA replication factor 1, SCF—Skp1-Cul1/Cdc53-F-box protein. Detailed explanations are provided in the text.

In somatic mammalian cells, G1 is the longest phase of the cell cycle because, during this phase, all regular activities of the cell and its organelles take place. Among them, there is a global increase in histone acetylation and transcriptional activity [31], potentially exposing large regions of the genome to unwarranted programmable nuclease-induced NHEJ during G1. The anaphase-promoting complex (APC) with the activator protein Cdh1 (APC-Cdh1) forms the E3 ubiquitin ligase complex, which is active in the late M and G1 phases of the cell cycle, timely triggering ubiquitination and ensuing proteasomal degradation of the target cell cycle proteins, including geminin (Figure 1) [32,33]. Gutschner et al. (2016) [34] proposed an elegant solution for cell cycle-dependent expression of Cas9—fusion of Cas9 with geminin—resulting in lower expression of Cas9 in G1 and higher expression in S/G2/M phases because of the activity of APC-Cdh1. Cas9-geminin fusion resulted in a 1.9-fold increase in knock-in at the *MYH7* locus in porcine fibroblast cultures [35] and a growth in the HDR efficiency at the *MALAT1* locus from 9.7% to 13.8% in HEK293T cells. The combination of this approach with nocodazole treatment in the case of the *MALAT1* locus led to an increase in the HDR rate to 16.2% [36]. It was shown that the NHEJ/HDR ratio significantly decreased independently of the chromatin structure when using geminin [37].

The authors of the cited studies did not note a cell apoptosis increase or geminin toxicity; moreover, Yang et al. noted that the Cas9-geminin fusion shortened the lifespan of Cas9 in the cell and thereby reduced its toxicity to mice neurons in vivo [38]. Many studies using geminin have shown that a fragment of the protein, 110 amino acids (aa) for human geminin [34,37] or even 40 aa for mouse geminin [38], is sufficient to control Cas9 expression in the S/G2/M phases.

A similar approach to the regulation of Cas9 expression in a cell cycle-dependent manner was proposed by Matsumoto et al. [39]. The researchers used AcrIIA4, a natural inhibitor of Cas9, which was fused with the human chromatin licensing and DNA replication factor 1 (hCdt1) (Figure 1). The AcrIIA4-hCdt1 complex inhibits Cas9 in the G1 phase; in the S/G2 phases, the complex undergoes proteolysis by the SCF-Skp2 complex and releases Cas9 activity. Indeed, this approach helped increase the HDR efficiency of CRISPR-Cas9 by 1.7–4.5 times, depending on the target locus.

Despite the fairly good results obtained with cell cycle-dependent degradation of molecules (geminin or Cdt1) in genome editing experiments, there is still little scientific research in this area, which is inexplicable.

## 4. Modulation of Cyclin-Dependent Kinases

**Figure 2:**
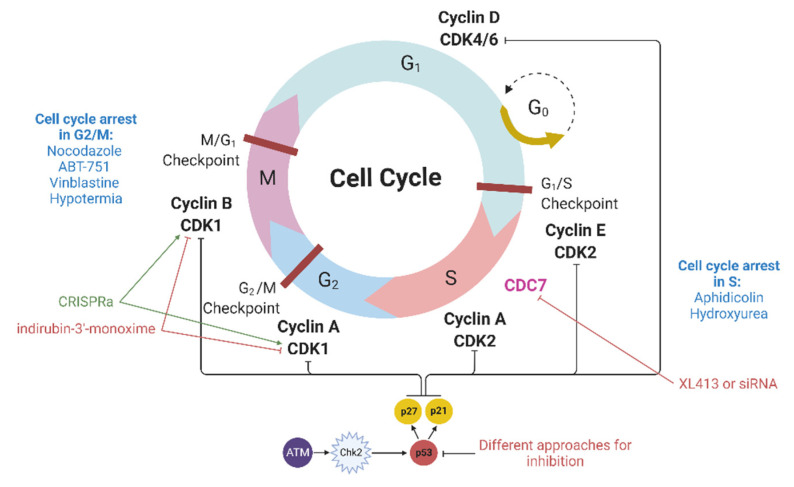
Mechanism of p53-mediated cell cycle arrest and possible approaches to influencing the cell cycle. DSBs activate ATM kinase and Chk2; this complex phosphorylates p53, which promotes the expression of p21 and p27. The latter binds and inhibits cyclin and cyclin-dependent kinase complexes, leading to cell cycle arrest in the G1/S phase as well as in the M/G1 and G2/M phases. ATM—serine/threonine kinase mutated in ataxia-telangiectasia, Chk2—checkpoint kinase 2. Detailed explanations are provided in the text.

Cyclin-dependent kinases (CDKs) are heterodimeric serine/threonine protein kinases that regulate cell cycle progression. Among them, the Cdk1-cyclin B complex controls the cell transition from the G2 to the M phase, while the Cdk2-cyclin E and Cdk2-cyclin A complexes regulate the G1/S and S/G2 transitions (Figure 2). In addition to CDKs, many CDK inhibitors (CDKIs), including members of the CIP/KIP family (p21, p27, and p57), are also involved in the regulation of the cell cycle. p21 interacts with a number of transcription factors, and the overexpression of p21 induces cell cycle arrest in various phases of the cell cycle [40].

Several cyclin-dependent kinases can be inhibited by various molecules to achieve cell cycle synchronization in the G1/S or G2/M phases, for example, by indirubins [41]. In *GFP* knock-in experiments using CRISPR-Cas9, treatment of porcine fetal fibroblasts with indirubin-3′-monoxime (4 µg/mL), an inhibitor of cyclin-dependent kinase 1 (CDK1), increased HDR by 1.9 times (up to 19.7%) [24]. Similar results were obtained for HeLa, HT-1080, and U-2 OS cells: an increase in the HDR rate by 2–5 times using transfection with expression vectors, coding meganuclease I-SceI, or ZFNs. In addition, in mesenchymal stem cells, indirubin-3′-monoxime also led to a 10-fold increase in HDR [21]. However, indirubins were found to increase apoptosis at almost all tested concentrations [42].

CDC7, a factor necessary to enter the S phase, can be indirectly included in this section. The CDC7 protein is involved in DNA replication; therefore, its inhibition leads to cell cycle arrest at the G1/S point [43,44]. The inhibition of CDC7 by XL413 led to a 2.1-fold increase in HDR in the K562 cell line, while its inhibition by siRNA increased HDR by 1.4 times [45]. Similar results were obtained in iPSCs: XL413 increased the HDR rate by 1.7 times (33.7% vs. 19.4%) during the integration of a *BFP* gene fragment into the *AAVS1-EGFP* locus. The combination of XL413 with the NHEJ inhibitors NU7441 and SCR7 further increased HDR by 2.7 times (45.7% vs. 17%) in iPSCs [44]. Importantly, XL413 must be added 24 h after CRISPR-Cas9 injection in order to achieve cell synchronization in the S phase [45].

As can be seen from the results of the studies presented above, both strategies (synchronizing cells in the G1/S or G2/M phases) ultimately lead to an increase in the efficiency of HDR/HR in genome editing experiments. This is probably due to the fact that the DSB repair pathway regulated by BRCA1/BRCA2, i.e., HDR/HR, is active in the S and G2 phases [12].

As stated above, CDK1 inhibition increases HDR; however, the opposite approach also enhances it. It has been shown that CDK1 promotes efficient end resection by phosphorylating the DSB resection nuclease, so its activation also contributes to an increase in HDR efficiency. CDK1 activation by CRISPRa increased HDR in HEK293, HEK293T, and HeLa cell cultures by 2.0–4.4 times (up to 7.58%), depending on the culture, locus of integration, and transgene. This activation had a synergistic effect (up to 15.3%) with the inhibition of KU80, a key factor in NHEJ, by CRISPRi [46].

Nonetheless, for unknown reasons, CDK inhibitors are rarely used to increase the efficiency of HDR in genome editing experiments despite the fact that several dozens of them have already been described, and many of them are commercially available [47].

## 5. Inhibition of p53

Nuclease-mediated DNA double-stranded breaks by themselves can also cause cell cycle arrest. If DNA double-stranded breaks occur, then ATM kinase is activated, which in turn activates Chk2 (Figure 2). Then, the ATM-Chk2 complex phosphorylates p53, which promotes p21 expression. The latter binds and inhibits cyclin and cyclin-dependent kinase complexes, leading to cell cycle arrest in the G1/S phase as well as in the M/G1 and G2/M phases [48]. Genome editing methods involve the production of DSBs and thereby can activate the ATM–Chk2 pathway, which may result in cell cycle arrest [49]. Even single nuclease-induced DSBs in hematopoietic stem and progenitor cells can activate the p53 pathway, although this phenomenon is reversible [50]. G2/M arrest is indicative of DNA damage, likely caused by the combined on- and off-target activities of the nucleases. However, when using highly specific methods, DSBs should not be generated in large quantities (their significant increase can be caused only by a high nonspecific activity); therefore, cell cycle arrest cannot be expected to occur in all cells. Nevertheless, we must be aware of its possibility.

The activation of p53 is accompanied by cell cycle arrest in the G1 phase [51,52]. As noted earlier, HDR does not occur in the G1 phase; therefore, the inhibition of p53 may help to increase HDR. Indeed, the efficiency of GFP restoration using CRISPR-Cas9 in p53-deficient RPE1 cells was significantly higher than in the cells with wild-type p53 [51]. Subsequently, this observation was confirmed by several independent scientific groups in human pluripotent [53] and hematopoietic stem cells [50], as well as in ductal and hepatocyte organoids [54]. The main explanation for this phenomenon is the activation of p53 by nuclease-induced double-stranded breaks, which leads to p53-dependent arrest [51]. Nevertheless, the genome-scale CRISPR screen in different cell lines showed that p53 activation does not occur in all cell cultures, which should be taken into account in experimental work [55].

It is known that p53 is a key factor causing cell apoptosis in cases of abnormalities [56]; therefore, temporary inhibition of p53 may be unsafe and may lead to the accumulation of DNA damage in the cell, which must be taken into account when using this approach. It has been proven that p53 activates p21, which in turn inhibits all CDKs and arrests the cell cycle in any phase (M/G1, G1/S, and G2/M) [48]. Another concern is the possible clonal expansion of cells with mutations in the *TP53* gene under conditions of in vivo genome editing, which could potentially lead to the development of tumors [57]. Recently, the selective advantage of cells with p53-inactivating mutations was experimentally confirmed in CRISPR-Cas9 studies [58].

## 6. Mitogens

Cell cycle synchronization can be achieved not only by the arrest of all cells in the culture at a certain point in the cell cycle but also by the simultaneous progression of the cycle for all available cells. This can be achieved with the help of mitogens, molecules that signal cells to enter the S phase [59].

**Figure 3:**
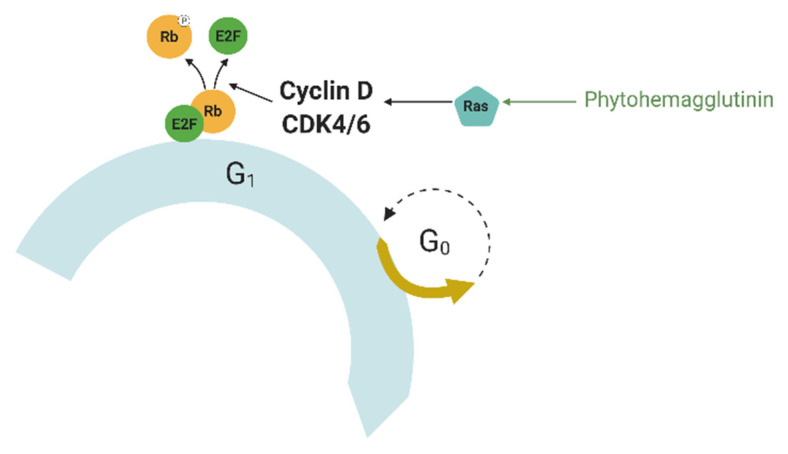
Mechanism of mitogen’s action on the cell cycle. The binding of a mitogen to its receptor induces the activation of the Ras protein, which leads to the activation of the expression of cyclin D, which forms a complex with Cdk4 or Cdk6 called the cyclin D-Cdk complex. This complex dissociates the Rb-E2F complex and phosphorylates the Rb protein, which controls the expression of a number of genes required for DNA replication and mitosis. Ras—rat sarcoma protein, Rb—retinoblastoma protein. Detailed explanations are provided in the text.

Mitogens are typically small proteins that act as a signal to start cell division. Some growth factors are mitogens, such as epidermal growth factor (EGF) [60] and platelet-derived growth factor (PDGF) [61], but others, such as vascular endothelial growth factor (VEGF), are not [62]. They act via mitogen-activated protein kinases (MAPKs) and lead to the induction of mitosis (MAPK signaling pathway). Once the cells pass the G1 checkpoint, which is controlled by mitogens, the latter are no longer needed to continue the progress through the cell cycle. The MAPK signaling pathway is implicated in many cancers because dysregulation of this pathway leads to uncontrolled growth [63,64]. Mammalian cells require mitogens to proliferate; mammalian cell membranes have mitogen receptors that are typically receptor-associated tyrosine kinases, such as the epidermal growth factor receptor (EGFR). The binding of a mitogen to its receptor induces the activation of the Ras (rat sarcoma) protein (Figure 3), which leads to the activation of gene expression through transcription factors, such as c-Myc, serum response factor, etc. [65]. They activate the expression of cyclin D, which forms a complex with Cdk4 or Cdk6 called cyclin D-Cdk complex. This complex phosphorylates the retinoblastoma protein (Rb). Phosphorylated Rb interacts with the transcription factor E2F, which controls the expression of a number of genes required for DNA replication and mitosis, such as cyclin A and cyclin E [66,67]. This is not the only control exerted by mitogens; they can inhibit the glycogen synthase kinase (GSK3β) via phosphoinositide 3-kinase. GSK3β is a kinase that phosphorylates cyclin D at Thr286, keeping the cyclin D-CDK complex present in the G0 phase inhibited [68]. Thus, mitogens can be used to force cells to simultaneously enter the S phase.

The use of mitogens to increase HDR in genome editing experiments is often limited only to phytohemagglutinin for editing T-lymphocytes. Phytohemagglutinin (PHA) is a lectin derived from red kidney bean extract. It has strong agglutinating and mitogenic activities. PHA has been used for T-cell activation since 1960 [69]. It is also actively used in protocols for karyotyping T-lymphocytes. Kuo et al. showed that the efficiency of integration of the *GFP* cDNA into the 5′-UTR of the *CD40LG* gene in Jurkat T-cells using TALEN increased with the addition of PHA in a dose-dependent manner up to 20.7% (with 3 μg/mL PHA) [70]. However, no other similar studies, either with PHA or with other mitogens, have been published to date.

The lack of studies using mitogens may indicate that researchers understand the likely negative consequences. Hypophosphorylated (inactive) Rb is one of the key factors that prevent cells with damaged DNA from proliferating. Thus, overactivation (due to phosphorylation) of Rb would result in the appearance of cells with damaged DNA, which is not acceptable in clinical applications of this approach. Because of this, the use of mitogens to increase genome editing efficiency can be a double-edged sword.

## 7. Other Factors Causing Cell Cycle Synchronization

### 7.1. Aphidicolin

Aphidicolin inhibits DNA polymerase-α and δ and blocks DNA synthesis, arresting the cell cycle in the S phase [71]. Studies have shown that aphidicolin leads to an increase in HDR efficiency in CRISPR-Cas9 experiments in primary neonatal fibroblast cell cultures by 1.3 times and in embryonic stem cells by 1.6 times [19]. In HCT116 cells, the efficiency of EGFP recovery using ssODN and CRISPR-Cas9 in synchronized cells was shown to increase by 4–5 times, depending on the number of the plasmid encoding CRISPR-Cas9 components [72]. After aphidicolin treatment, embryonic stem cells retained their pluripotency [19].

### 7.2. Hydroxyurea

Hydroxyurea (HU) inhibits ribonucleotide reductase and arrests pro- and eukaryotic cells in the S phase [73,74], which can be useful for HDR enhancement in genome editing experiments. A simple addition of HU (2 mM) did not increase the frequency of insertion of the CMV and hEF1α promoters into the selected genome locus using CRISPR-Cas9 in CHO cells [74]. However, with additional cell selection in the presence of HU (0.125 mM), it increased by 1.2–1.5 times. At low concentrations, HU did not increase apoptosis [74].

HU (100 µM) also increased HDR in the K562 cell line, in which the coding sequence of the *EGFP* gene was inserted into the *Gγ-globin* locus; however, the extent of increase was difficult to determine because the cell clones with the insert were subjected to additional G418 selection [75].

### 7.3. Hypothermia

It was previously shown that hypothermia can induce reversible p53-mediated cell cycle arrest in some cells [76,77]. Maurissen et al. showed that culturing iPSCs at 32 °C for 48 h increased the proportion of cells in the G2/M phase to 25.5%, compared with 6.0% in the control conditions, and suppressed DNA synthesis. Mild hypothermia increased HDR by 1.4 times (30.1% vs. 21.3%) in iPSCs when a *BFP* gene fragment was integrated into the *AAVS1-EGFP* locus. The combination of hypothermia with NHEJ inhibitors (NU7441 and SCR7) additionally increased HDR up to 48.9%, while the combination with XL413 increased HDR by 2 times [44].

## 8. Conclusions

Genome editing for the treatment of human diseases can be implemented both in the form of gene therapy (when delivered in vivo) and in the form of gene-cell therapy (ex vivo). In the latter case, the use of factors that influence the cell cycle and make it possible to enrich the cell population at certain phases of the cell cycle will increase the efficiency of the insertion of genes or their fragments into the desired locus of the genome. This, in turn, can reduce the time and cost of producing a modified cell product. Meanwhile, studies have shown that a number of FDA-approved anticancer drugs (such as vinblastine and hydroxyurea) also increase the knock-in frequency, which could potentially be used for in vivo editing. If genome editing is carried out for ex vivo treatment and researchers have the opportunity to select successfully edited cells, then the approaches described in this article are not practical. However, when it comes to ex vivo therapy without cell selection and especially in vivo therapy, highly efficient HDR may be required.

Certainly, additional research is needed, but it can already be argued that some approaches allow an increase in the efficacy of HDR in genome editing experiments by several times. These approaches include the use of nocodazole for cell synchronization in the G2/M phase, the use of geminin for controlled expression of Cas9 at the desired phase of the cell cycle, a combination of different factors, etc. At the same time, it is necessary to use caution in the use of mitogens and the inhibition of p53 because of the possible accumulation of DNA damage in the cell. The transplantation of the resulting edited cells may have severe negative consequences; therefore, thorough and comprehensive studies of the effects of this approach are necessary. Any interference with the cell cycle can potentially lead to increased apoptosis or uncontrolled cell proliferation. In humans, cell cycle arrest in the G1, G2, and M phases potentially leads to cell apoptosis, whereas cell synchronization in the S phase leads to cell proliferation. Despite the fact that many substances that cause cell cycle arrest are toxic (causing apoptosis, for example), cells often return to normal morphology and vital activity after the withdrawal of these substances, that is, the drugs have a reversible effect, even in the case of stem cells. Therefore, in our opinion, such edited cells can be used for ex vivo therapy. A different situation occurs with in vivo therapy, in which such substances act on the cells of the whole organism, leading to unwanted apoptosis in various organs and tissues. Of course, prescribing even FDA-approved drugs in this case should be accompanied by an assessment of the potential benefits and risks.

On the other hand, the development of biotechnology makes it possible, in some cases, to carry out the editing of mutations without the use of HDR; modifications of CRISPR-Cas9, base editing (BE) [78], and prime editing (PE) [79] enable this. These methods are based on the use of a mutant form of nuclease that is capable of cutting only one DNA strand (nickase) or is completely devoid of activity (dead Cas9). Since a DNA double-stranded break is not created in this case, nonspecific activity both at the targeted and non-targeted loci is significantly reduced, and the efficiency of knock-in is often higher due to the fact that DNA repair occurs via other pathways (base excision repair for BE or mismatch repair for PE) that are active throughout the cell cycle. Proteins fused with inactivated Cas9, deaminase (in BE), and reverse transcriptase (in PE) provide direct or indirect DNA editing. Deaminase can convert cytosine to thymine [78] or adenine to guanine [80] at the target DNA locus, while reverse transcriptase is capable of synthesizing a donor molecule to repair a DNA break [79]. BE and PE have already shown high efficiency and low incidence of side effects, which brings us closer to the introduction of these methods into clinical practice.

## Figures and Tables

**Table 1:** Impact of microtubule inhibitors on HDR.

Cell Line/Culture	Microtubule Inhibitor	Concentration	Effect on HDR	Reference
HEK293T	Nocodazole	200 ng/mL	Up to 6-fold increase	[19]
HEK293T	Nocodazole	100 ng/mL	None	[20]
HeLa	Vinblastine	40 nM	7-fold increase	[21]
Induced pluripotent stem cells (iPSCs)	Nocodazole	100 ng/mL	1.7-fold increase	[22]
Human pluripotent stem cells (hPSCs)	Nocodazole	1 µg/mL	3.5-fold increase without cell selection	[23]
Human pluripotent stem cells (hPSCs)	ABT-751	0.37 μg/mL	3.1-fold increase	[23]
Umbilical cord-derived mesenchymal stem cells	Vinblastine	40 nM	None	[21]
Porcine fetal fibroblasts	Nocodazole	1 µg/mL	2.8-fold increase in HDR	[24]

## Data Availability

Not applicable.

## References

[1] Lombardo A., Genovese P., Beausejour C.M., Colleoni S., Lee Y.L., Kim K.A., Ando D., Urnov F.D., Galli C., Gregory P.D. (2007). Gene editing in human stem cells using zinc finger nucleases and integrase-defective lentiviral vector delivery. Nat. Biotechnol..

[2] Zou J., Maeder M.L., Mali P., Pruett-Miller S.M., Thibodeau-Beganny S., Chou B.K., Chen G., Ye Z., Park I.H., Daley G.Q. (2009). Gene targeting of a disease-related gene in human induced pluripotent stem and embryonic stem cells. Cell Stem Cell.

[3] Hockemeyer D., Wang H., Kiani S., Lai C.S., Gao Q., Cassady J.P., Cost G.J., Zhang L., Santiago Y., Miller J.C. (2011). Genetic engineering of human pluripotent cells using TALE nucleases. Nat. Biotechnol..

[4] Zhang F., Wen Y., Guo X. (2014). CRISPR/Cas9 for genome editing: Progress, implications and challenges. Hum. Mol. Genet..

[5] Haber J.E. (2000). Partners and pathways repairing a double-strand break. Trends Genet..

[6] Lieber M.R. (2008). The mechanism of human nonhomologous DNA end joining. J. Biol. Chem..

[7] Thompson L.H., Schild D. (2001). Homologous recombinational repair of DNA ensures mammalian chromosome stability. Mutat. Res..

[8] Barnum K.J., O’Connell M.J. (2014). Cell cycle regulation by checkpoints. Methods Mol. Biol..

[9] Lieber M.R. (2010). The mechanism of double-strand DNA break repair by the nonhomologous DNA end-joining pathway. Annu. Rev. BioChem..

[10] Takata M., Sasaki M.S., Sonoda E., Morrison C., Hashimoto M., Utsumi H., Yamaguchi-Iwai Y., Shinohara A., Takeda S. (1998). Homologous recombination and non-homologous end-joining pathways of DNA double-strand break repair have overlapping roles in the maintenance of chromosomal integrity in vertebrate cells. EMBO J..

[11] Arnoult N., Correia A., Ma J., Merlo A., Garcia-Gomez S., Maric M., Tognetti M., Benner C.W., Boulton S.J., Saghatelian A. (2017). Regulation of DNA repair pathway choice in S and G2 phases by the NHEJ inhibitor CYREN. Nature.

[12] Smirnikhina S.A., Anuchina A.A., Lavrov A.V. (2019). Ways of improving precise knock-in by genome-editing technologies. Hum. Genet..

[13] Jordan M.A. (2002). Mechanism of action of antitumor drugs that interact with microtubules and tubulin. Curr. Med. Chem. Anticancer Agents.

[14] Downing K.H., Nogales E. (1998). Tubulin and microtubule structure. Curr. Opin. Cell Biol..

[15] Nogales E. (2000). Structural insights into microtubule function. Annu. Rev. BioChem..

[16] Silva P., Barbosa J., Nascimento A.V., Faria J., Reis R., Bousbaa H. (2011). Monitoring the fidelity of mitotic chromosome segregation by the spindle assembly checkpoint. Cell Prolif..

[17] Carlomagno T. (2009). Tubulin-binding Agents: Synthetic, Structural and Mechanistic Insights.

[18] Hamel E., Fojo T. (2008). An Overview of Compounds That Interact with Tubulin and Their Effects on Microtubule Assembly. The Role of Microtubules in Cell Biology, Neurobiology, and Oncology.

[19] Lin S., Staahl B.T., Alla R.K., Doudna J.A. (2014). Enhanced homology-directed human genome engineering by controlled timing of CRISPR/Cas9 delivery. Elife.

[20] Yan N., Sun Y., Fang Y., Deng J., Mu L., Xu K., Mymryk J.S., Zhang Z. (2020). A Universal Surrogate Reporter for Efficient Enrichment of CRISPR/Cas9-Mediated Homology-Directed Repair in Mammalian Cells. Mol. Ther. Nucleic Acids.

[21] Rahman S.H., Bobis-Wozowicz S., Chatterjee D., Gellhaus K., Pars K., Heilbronn R., Jacobs R., Cathomen T. (2013). The nontoxic cell cycle modulator indirubin augments transduction of adeno-associated viral vectors and zinc-finger nuclease-mediated gene targeting. Hum. Gene Ther..

[22] Zhang J.P., Li X.L., Li G.H., Chen W., Arakaki C., Botimer G.D., Baylink D., Zhang L., Wen W., Fu Y.W. (2017). Efficient precise knockin with a double cut HDR donor after CRISPR/Cas9-mediated double-stranded DNA cleavage. Genome Biol..

[23] Yang D., Scavuzzo M.A., Chmielowiec J., Sharp R., Bajic A., Borowiak M. (2016). Enrichment of G2/M cell cycle phase in human pluripotent stem cells enhances HDR-mediated gene repair with customizable endonucleases. Sci. Rep..

[24] Xie Z., Pang D., Wang K., Li M., Guo N., Yuan H., Li J., Zou X., Jiao H., Ouyang H. (2017). Optimization of a CRISPR/Cas9-mediated Knock-in Strategy at the Porcine Rosa26 Locus in Porcine Foetal Fibroblasts. Sci. Rep..

[25] Wang Y., Zhang H., Gigant B., Yu Y., Wu Y., Chen X., Lai Q., Yang Z., Chen Q., Yang J. (2016). Structures of a diverse set of colchicine binding site inhibitors in complex with tubulin provide a rationale for drug discovery. FEBS J..

[26] La Regina G., Coluccia A., Naccarato V., Silvestri R. (2019). Towards modern anticancer agents that interact with tubulin. Eur. J. Pharm. Sci..

[27] Baldin V., Lukas J., Marcote M.J., Pagano M., Draetta G. (1993). Cyclin D1 is a nuclear protein required for cell cycle progression in G1. Genes Dev..

[28] Yoshimatsu K., Yamaguchi A., Yoshino H., Koyanagi N., Kitoh K. (1997). Mechanism of action of E7010, an orally active sulfonamide antitumor agent: Inhibition of mitosis by binding to the colchicine site of tubulin. Cancer Res..

[29] Hande K.R., Hagey A., Berlin J., Cai Y., Meek K., Kobayashi H., Lockhart A.C., Medina D., Sosman J., Gordon G.B. (2006). The pharmacokinetics and safety of ABT-751, a novel, orally bioavailable sulfonamide antimitotic agent: Results of a phase 1 study. Clin. Cancer Res..

[30] Moudi M., Go R., Yien C.Y., Nazre M. (2013). Vinca alkaloids. Int. J. Prev. Med..

[31] Bou Kheir T., Lund A.H. (2010). Epigenetic dynamics across the cell cycle. Essays BioChem..

[32] Almeida A., Bolaños J.P., Moncada S. (2010). E3 ubiquitin ligase APC/C-Cdh1 accounts for the Warburg effect by linking glycolysis to cell proliferation. Proc. Natl. Acad. Sci. USA.

[33] Rizzardi L.F., Cook J.G. (2012). Flipping the switch from g1 to s phase with e3 ubiquitin ligases. Genes Cancer.

[34] Gutschner T., Haemmerle M., Genovese G., Draetta G.F., Chin L. (2016). Post-translational Regulation of Cas9 during G1 Enhances Homology-Directed Repair. Cell Rep..

[35] Howden S.E., McColl B., Glaser A., Vadolas J., Petrou S., Little M.H., Elefanty A.G., Stanley E.G. (2016). A Cas9 Variant for Efficient Generation of Indel-Free Knockin or Gene-Corrected Human Pluripotent Stem Cells. Stem Cell Reports.

[36] Gerlach M., Kraft T., Brenner B., Petersen B., Niemann H., Montag J. (2018). Efficient Knock-in of a Point Mutation in Porcine Fibroblasts Using the CRISPR/Cas9-GMNN Fusion Gene. Genes.

[37] Janssen J.M., Chen X., Liu J., Gonçalves M.A.F.V. (2019). The Chromatin Structure of CRISPR-Cas9 Target DNA Controls the Balance between Mutagenic and Homology-Directed Gene-Editing Events. Mol. Ther. Nucleic Acids.

[38] Yang S., Li S., Li X.J. (2018). Shortening the Half-Life of Cas9 Maintains Its Gene Editing Ability and Reduces Neuronal Toxicity. Cell Rep..

[39] Matsumoto D., Tamamura H., Nomura W. (2020). A cell cycle-dependent CRISPR-Cas9 activation system based on an anti-CRISPR protein shows improved genome editing accuracy. Commun. Biol..

[40] Huang B., Mu P., Chen X., Tang S., Ye W., Zhu W., Deng Y. (2019). Aflatoxin B1 induces S phase arrest by upregulating the expression of p21 via MYC, PLK1 and PLD1. Biochem. Pharmacol..

[41] Eisenbrand G., Hippe F., Jakobs S., Muehlbeyer S. (2004). Molecular mechanisms of indirubin and its derivatives: Novel anticancer molecules with their origin in traditional Chinese phytomedicine. J. Cancer Res. Clin. Oncol..

[42] Shi J., Shen H.M. (2008). Critical role of Bid and Bax in indirubin-3′-monoxime-induced apoptosis in human cancer cells. Biochem. Pharmacol..

[43] Moiseeva T.N., Bakkenist C.J. (2018). Regulation of the initiation of DNA replication in human cells. DNA Repair.

[44] Maurissen T.L., Woltjen K. (2020). Synergistic gene editing in human iPS cells via cell cycle and DNA repair modulation. Nat. Commun..

[45] Wienert B., Nguyen D.N., Guenther A., Feng S.J., Locke M.N., Wyman S.K., Shin J., Kazane K.R., Gregory G.L., Carter M.A.M. (2020). Timed inhibition of CDC7 increases CRISPR-Cas9 mediated templated repair. Nat. Commun..

[46] Ye L., Wang C., Hong L., Sun N., Chen D., Chen S., Han F. (2018). Programmable DNA repair with CRISPRa/i enhanced homology-directed repair efficiency with a single Cas9. Cell Discov..

[47] Law M.E., Corsino P.E., Narayan S., Law B.K. (2015). Cyclin-Dependent Kinase Inhibitors as Anticancer Therapeutics. Mol. Pharmacol..

[48] Gomez V., Hergovich A. (2016). Cell-Cycle Control and DNA-Damage Signaling in Mammals. Genome Stability.

[49] Händel E.M., Gellhaus K., Khan K., Bednarski C., Cornu T.I., Müller-Lerch F., Kotin R.M., Heilbronn R., Cathomen T. (2012). Versatile and efficient genome editing in human cells by combining zinc-finger nucleases with adeno-associated viral vectors. Hum. Gene Ther..

[50] Schiroli G., Conti A., Ferrari S., Della Volpe L., Jacob A., Albano L., Beretta S., Calabria A., Vavassori V., Gasparini P. (2019). Precise Gene Editing Preserves Hematopoietic Stem Cell Function following Transient p53-Mediated DNA Damage Response. Cell Stem Cell.

[51] Haapaniemi E., Botla S., Persson J., Schmierer B., Taipale J. (2018). CRISPR-Cas9 genome editing induces a p53-mediated DNA damage response. Nat. Med..

[52] Geisinger J.M., Stearns T. (2020). CRISPR/Cas9 treatment causes extended TP53-dependent cell cycle arrest in human cells. Nucleic Acids Res..

[53] Ihry R.J., Worringer K.A., Salick M.R., Frias E., Ho D., Theriault K., Kommineni S., Chen J., Sondey M., Ye C. (2018). p53 inhibits CRISPR-Cas9 engineering in human pluripotent stem cells. Nat. Med..

[54] Artegiani B., Hendriks D., Beumer J., Kok R., Zheng X., Joore I., Chuva de Sousa Lopes S., van Zon J., Tans S., Clevers H. (2020). Fast and efficient generation of knock-in human organoids using homology-independent CRISPR-Cas9 precision genome editing. Nat. Cell Biol..

[55] Brown K.R., Mair B., Soste M., Moffat J. (2019). CRISPR screens are feasible in TP53 wild-type cells. Mol. Syst. Biol..

[56] Gottlieb T.M., Oren M. (1998). p53 and apoptosis. Semin. Cancer Biol..

[57] Foronda M., Dow L.E. (2018). CRISPR: Stressed about p53?. Trends Mol. Med..

[58] Enache O.M., Rendo V., Abdusamad M., Lam D., Davison D., Pal S., Currimjee N., Hess J., Pantel S., Nag A. (2020). Cas9 activates the p53 pathway and selects for p53-inactivating mutations. Nat. Genet..

[59] Hume S., Dianov G.L., Ramadan K. (2020). A unified model for the G1/S cell cycle transition. Nucleic Acids Res..

[60] Zeng F., Harris R.C. (2014). Epidermal growth factor, from gene organization to bedside. Semin Cell Dev. Biol..

[61] Alvarez R.H., Kantarjian H.M., Cortes J.E. (2006). Biology of platelet-derived growth factor and its involvement in disease. Mayo Clin. Proc..

[62] Kajdaniuk D., Marek B., Borgiel-Marek H., Kos-Kudła B. (2011). Vascular endothelial growth factor (VEGF)—Part 1: In physiology and pathophysiology. Endokrynol. Pol..

[63] Fecher L.A., Amaravadi R.K., Flaherty K.T. (2008). The MAPK pathway in melanoma. Curr. Opin. Oncol..

[64] Fang J.Y., Richardson B.C. (2005). The MAPK signalling pathways and colorectal cancer. Lancet Oncol..

[65] Martinelli E., Morgillo F., Troiani T., Ciardiello F. (2017). Cancer resistance to therapies against the EGFR-RAS-RAF pathway: The role of MEK. Cancer Treat. Rev..

[66] Johnson D.G., Schneider-Broussard R. (1998). Role of E2F in cell cycle control and cancer. Front. Biosci..

[67] Giacinti C., Giordano A. (2006). RB and cell cycle progression. Oncogene.

[68] Diehl J.A., Cheng M., Roussel M.F., Sherr C.J. (1998). Glycogen synthase kinase-3beta regulates cyclin D1 proteolysis and subcellular localization. Genes Dev..

[69] Nowell P.C. (1960). Phytohemagglutinin: An initiator of mitosis in cultures of normal human leukocytes. Cancer Res..

[70] Kuo C.Y., Long J.D., Campo-Fernandez B., de Oliveira S., Cooper A.R., Romero Z., Hoban M.D., Joglekar A.V., Lill G.R., Kaufman M.L. (2018). Site-Specific Gene Editing of Human Hematopoietic Stem Cells for X-Linked Hyper-IgM Syndrome. Cell Rep..

[71] Pérez-Benavente B., Farràs R. (2016). Cell Synchronization Techniques to Study the Action of CDK Inhibitors. Methods Mol. Biol..

[72] Bialk P., Sansbury B., Rivera-Torres N., Bloh K., Man D., Kmiec E.B. (2016). Analyses of point mutation repair and allelic heterogeneity generated by CRISPR/Cas9 and single-stranded DNA oligonucleotides. Sci. Rep..

[73] Tsakraklides V., Brevnova E., Stephanopoulos G., Shaw A.J. (2015). Improved Gene Targeting through Cell Cycle Synchronization. PLoS ONE.

[74] Kwak J.M., Lee Y., Shin S.W., Lee J.S. (2021). Hydroxyurea selection for enhancement of homology-directed targeted integration of transgenes in CHO cells. N Biotechnol..

[75] Jafari H., Hesami S., Safi M., Ghasemi F., Banan M. (2019). Expression and hydroxyurea-triggered induction of EGFP upon CRISPR/Cas9-mediated integration into the γ-globin gene of K562 cells. Biotechnol. Lett..

[76] Roobol A., Carden M.J., Newsam R.J., Smales C.M. (2009). Biochemical insights into the mechanisms central to the response of mammalian cells to cold stress and subsequent rewarming. FEBS J..

[77] Matijasevic Z., Snyder J.E., Ludlum D.B. (1998). Hypothermia causes a reversible, p53-mediated cell cycle arrest in cultured fibroblasts. Oncol. Res..

[78] Komor A.C., Kim Y.B., Packer M.S., Zuris J.A., Liu D.R. (2016). Programmable editing of a target base in genomic DNA without double-stranded DNA cleavage. Nature.

[79] Anzalone A.V., Randolph P.B., Davis J.R., Sousa A.A., Koblan L.W., Levy J.M., Chen P.J., Wilson C., Newby G.A., Raguram A. (2019). Search-and-replace genome editing without double-strand breaks or donor DNA. Nature.

[80] Gaudelli N.M., Komor A.C., Rees H.A., Packer M.S., Badran A.H., Bryson D.I., Liu D.R. (2017). Programmable base editing of A•T to G•C in genomic DNA without DNA cleavage. Nature.

